# Provident Medical Institution of Scotland

**Published:** 1830-04-01

**Authors:** 


### THE MEDICAL PROVIDENT INSTITUTION OF SCOTLAND.

Men, in all classes of life, are now rapidly awakening to the utility—we might say the necessity, of dedicating a pittance of their annual income to some fund or institution that may return a proportionate remuneration to their successors, in case of death, whether sudden or at the natural period. The advantages of assurance societies are now almost universally appreciated—and there is no class of society that ought more sedulously to attend to this species of provision for their families than medical men. The business of a merchant, of a tradesman, of a banker, may be carried on during bad health of the principal, or even after his decease; but with the medical man's death, his revenue instantly ceases—and even his temporary illness cuts off his income, in a great degree, for the time it lasts. Every thing depends on his personal ability to meet the incessant calls of the public—and a very short suspension of his functions not only detracts a corresponding portion of his revenue, but injures materially the income of succeeding years, however firmly his health may be re-established. This being the case, we are rejoiced to observe the growth and prosperity of a Provident Institution in North Britain, whose principal object appears to be that which is not contemplated in other institutions—a provision for the day of sickness.

"The objects of this Institution are generally—To protect the members throughout their whole lives, and to make provision for their widows, children, or other dependents, after their death. The casualties to which professional men are exposed, are—First, occasional or temporary inability to continue the personal exertions upon which their incomes depend, by reason of sickness or accident; and, second, The more durable or permanent incapacity arising from the infirmities of old age.

"To provide against these, one must either of himself accumulate funds during health and middle age, or join with others in raising a common fund, sufficient to protect those on whom the lot of suffering may fall—the former is not always in the power of professional men, even though their health and vigour should remain unimpaired to a late period of life—the latter has not been acted upon to the extent which the case demands—indeed, so far as relates to a period of incapacity from sickness or accident, the necessary protection *is unattainable in any other Institution.*

"In addition to the dangers to which medical men are subject during their own lives, it is scarcely necessary to add, that their widows, children, and other dependents, must be too often left in a destitute situation, rendered more painful, perhaps, by a few years of prosperity, and by the hopes it has led them to form.

"In no other Institution, accessible to the middle classes of society, with which we are acquainted, are funds established to meet all these contingencies. It was the principal object of the promoters of the Medical Provident Institution to supply this defect. Its scheme embraces the following benefits —I. Health Assurance—II. Deferred Annuities, and III. Contingent Annuities.

"I. *Health Assurance.* Under this head, provision is made for Professional incapacity, whether arising from sickness or accident, and this is combined with a permanent annuity in old age.

"The opportunity of making such a provision will appear in an especial manner highly beneficial to the country practitioner, when we bear in mind the many casualties to which he, in particular, is so frequently exposed in the exercise of his professional duties during long and weary journeys, often in dark and wintry nights, and over almost impassable roads—and to all medical men, when we consider that they have it least in their power to delegate their duties.

"It has been objected to this part of the scheme, that the institution will be liable to imposition, it being impossible in every case to ascertain the truth of the statements of members, especially those residing at a distance, and in the country. Independent, however, of the honourable feeling which we believe to exist throughout the profession, the institution, we think, will have ample security in the fact, that no member can have claim to this benefit, 'till five years after his entry, nor at any time for less than two weeks' illness, nor for more than two years, or 104 weeks in all. Should the disability, whether occurring at different periods, or continuing without intermission, exceed two years'—the member is then placed on the old age annuity, which is only one-half of the sick allowance. With such a limitation in view, it can never be the interest of any member to feign sickness; but, on the contrary, rather to save his allowances in middle age, and reserve them as a sure resource during the illness of a more advanced period. Should these be deemed insufficient securities, the fear of detection must ever prove a powerful, if not a complete check—it being one of the conditions of the Institution, that 'the policies become void if the persons assured shall be proved to have made any false statement, or concealed the truth at the time of their entering the Institution, *and if they shall practise imposition afterwards, by feigning indisposition or otherwise.*' Members who assure under this head forfeit the benefits also, if their illness has been occasioned by their own misconduct.

"II. *Deferred Annuities*—or annuities in old age, form the second table in the scheme. These annuities are combined with the health assurance in the former table; but they may also be assured separately, and may be entered upon at 50, 55, or 60: there is also a payment equal to a whole year's annuity, within three months after death.

"III. *Contingent Annuities*—or annuities to widows or other survivors, form the third table. These annuities contingent on the wife surviving the husband, are too well known to require any description here. They may be granted to others than the wives of members, such as brothers, sisters, or other nominees.

"The Medical Provident Institution is a *mutual* assurance scheme, and the whole funds belong to the assured; and should the rates of contribution be found to be higher than they might have been, the surplus will, in one form or other, be made available to the members, and not carried off by any body of proprietors."

We have not room for the numerical tables; but the following examples will sufficiently elucidate the ratio of subscription, and the advantages which are ultimately to result from it.

EXAMPLE I.

"A person of the age of 25, by a payment of 56*l*: 7 : 5 in one sum, or of 3*l*: 7 : 10 annually till he attains the age of 60, will be entitled to the following benefits:

1*st*, 1*l* per week while he is incapacitated by sickness, or other personal disability, from following his profession.

2*d*, An Annuity of 26*l*, to be entered upon when he attains the age of 60, and to be continued for life, payable half-yearly down to the day of his death.

3*d*, The sum of 26*l*. to his heirs, or others, as he may direct, payable within *three* months after his death."

EXAMPLE II.

"A person of the age of 30, by a single payment of 50*l*. 11s. or an annual payment of 3*l*: 5 : 2, from that age till 60, will be entitled to the following benefits:

1*st*, An Annuity of 25*l* for life, to be entered upon when he attains the age of 60, payable half-yearly, and down to the day of his death.

2*d*, The sum of 25*l* will be payable to his heirs, or others, as he may direct, within *three* months after his death.

EXAMPLE III.

"A Husband, by a single payment of 104*l*, or an annual payment of 8*l*: 13 : 8, during the joint lives of himself, aged 30, and his wife, aged 20, will entitle her, in the event of her surviving him, to an annuity of 25*l*. for her life, payable half-yearly, and down to the day of her death; with 25*l*. more, payable to her heirs, or others, as she may direct, within *three* months after death."

The list of directors embraces many of the first medical men in Scotland, and from the secretary, Mr. David Cannan, 41, Northumberland-street, Edinburgh, all necessary particulars may be learnt. We wish the Institution every success.

